# Hypomethylating Agents and Venetoclax Based Triplets Targeting FLT3, IDH and KMT2A in Acute Myeloid Leukemia: Current Studies and Challenges of a Tailored Approach

**DOI:** 10.3390/cancers18040615

**Published:** 2026-02-13

**Authors:** Elisa Santambrogio, Alessia Castellino, Ernesta Audisio, Martin Schumacher, Georg Feldmann, Raheel Iftikhar, Peter Brossart, Semra Aydin

**Affiliations:** 1 Division of Hematology and Allogeneic Stem Cell Transplant Unit, A.O.U. Città della Salute e della Scienza di Torino, 10126 Turin, Italy; 2 Department of Hematology, AO Santa Croce e Carle, 12100 Cuneo, Italy; castellino.ale@ospedale.cuneo.it; 3 Department of Hematology, Oncology, Rheumatology, Immunoncology and Stem-Cell Transplantation, University Hospital Bonn, Venusberg-Campus 1, 53127 Bonn, Germanypeter.brossart@ukbonn.de (P.B.); 4 Department of Hematology and Stem Cell Transplant, Armed Forces Bone Marrow Transplant Center/National Institute of Blood and Marrow Transplant, Rawalpindi 46000, Pakistan

**Keywords:** acute myeloid leukemia, triplets, hypomethylating agents, venetoclax

## Abstract

Recent implementations with novel target drugs of the hypomethylating agent/venetoclax doublet challenge our treatment approach in acute myeloid leukemia patients ineligible for intensive chemotherapy. Given the doublets’ efficacy, associations of agents based on the disease’s biology to the doublet backbone are leading to novel triplet (or more) combinations. In the present review mainly FLT3, IDH and KMT2A are discussed as possible targets in this context. These triplets do not only have efficacy in relapsed/refractory patients but also in treatment-naïve patients. Results from concluded and ongoing clinical trials, as well as real-world experiences, report high efficacies competing with intensive chemotherapy. For instance, the azacytidine/venetoclax/gilteritinib triplet as first-line is reported to induce a complete remission rate with and without incomplete recovery (CR/CRi) of 96%, with 90% of responders achieving minimal residual negativity. Once a stable CR was obtained, 47% of patients who were initially considered too frail for intensive chemotherapy were able to undergo allogeneic stem cell transplantation. However, there are still open questions and challenges regarding toxicity, post-remission therapy, and overall treatment duration. The present review will not only present the specific potency of these arising triplets, but also discuss their challenges and limitations, based on currently available data. Besides regimens containing approved inhibitors, triplets with next-generation inhibitors, including completely orally administered triplet regimens, are also summarized. Their promising results are leading to advanced phase clinical studies by international consortia and collaborative groups, aiming to further refine their clinical management.

## Simple Summary

The outcome of acute myeloid leukemia patients ineligible for intensive chemotherapy has dramatically improved by the implementation of the hypomethylating agent/venetoclax doublet. The therapeutic landscape is rapidly evolving, in both relapse/refractory and newly diagnosed settings. Driven by disease-specific features, personalised treatment strategies are developing through the incorporation of novel agents into the doublet backbone. This review aims to present and summarise the main data from recent clinical trials investigating these arising triplets, and, above all, addresses their main challenges and limitations based on currently available evidence. Given the broad spectrum of targets, triplets addressing FLT3, IDH, and KMT2A are chosen exemplarily.

## 1. Introduction

The oral B-cell lymphoma 2 (BCL-2) protein inhibitor venetoclax (VEN) is a key regulator of mitochondrial apoptosis [1]. Venetoclax induces cell death in acute myeloid leukemia (AML) cell lines, leukemic blasts, progenitor cells and stem cells of AML patients [2,3]. Azacitidine (AZA) inhibits the expression of pro-survival proteins, namely Myeloid Cell Leukemia 1 (MCL1) and BCL-XL, thus increasing the dependence of leukemia cells on BCL-2. In pre-clinical studies, the combination of AZA with VEN has been shown to induce cell death in AML-derived cell lines [4]. Their synergistic action has been confirmed in the phase-3 VIALE-A trial comparing AZA/VEN with AZA/placebo in 431 newly diagnosed (ND) AML patients [5] and resulting in higher complete remission (CR) rates (66.4% versus 28.3%) and overall survival (OS). The trial has been recently updated at a median follow-up of 43.2 months, confirming the superior median OS with 14.7 versus 9.6 months, respectively, *p* < 0.001 [6]. Based on these results, the hypomethylating agent (HMA)/VEN doublet has become the new standard for elderly AML patients ineligible for intensive induction chemotherapy. However, without consolidating allogeneic transplant, the AZA/VEN doublet therapy needs to be continued indefinitely [7,8]. This less-intensive treatment approach led to the implementation of newly defined risk categories. Successive molecular analysis of the VIALE-A cohort by Döhner and colleagues revealed certain limitations of the European Leukemia Network (ELN) 2017 and 2022 risk classifications and highlighted that distinct molecular subgroups benefited differentially from the doublet [9]. The presence of K/NRAS or FLT3-internal tandem domain (ITD) mutations was associated with an inferior median OS of 12.1 months compared to 26.5 months in wild-type (wt) patients. In the case of the TP53 mutation, median OS was dismal with 5.5 months. Complete remission rates after AZA/VEN ranged from 47.6% in the low-benefit (TP53 mutated) to 77.2% in the high-benefit (TP53-wt/K/NRAS-wt/FLT3-wt) subgroups. Not only significantly higher CR rates but also higher minimal residual disease (MRD) negativity rates were achieved across all groups, underlining the high therapeutic potential of the HMA/VEN combination [9]. Nevertheless, an improvement is needed, especially for the less benefiting groups, with the presence of K/NRAS, TP53 or FLT3-ITD, as also confirmed independently by Gangat and colleagues [10].

**Figure 1:**
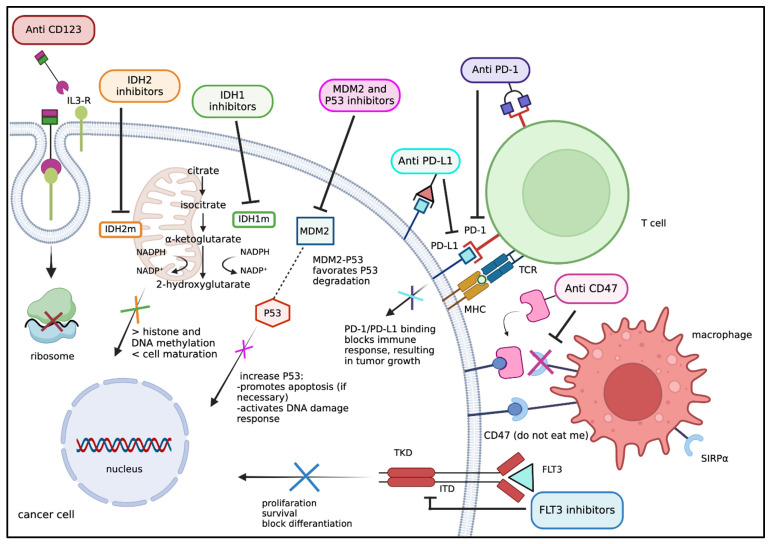
Drug mechanisms of action and possible targets. The presented targets are reported to be addressed in possible HMA/VEN-based triplets. FLT3: FMS-like tyrosine kinase-3; IDH1/2: isocitrate dehydrogenase 1/2; IDH1m: mutated isocitrate dehydrogenase 1; IDH2m: mutated isocitrate dehydrogenase 2; IL-3 R: interleukin-3 receptor; ITD: internal tandem duplication; MDM2: mouse double minute 2; MHC: major histocompatibility complex; PD-1: programmed death-1; PD-L1: programmed death-ligand 1; SIRP α: signal regulatory protein α; TCR: T-cell receptor; TKD: tyrosine kinase domain.

Promising investigational approaches, such as adding additional targeted therapies to the HMA/VEN backbone, are currently leading to novel, individualised triplet (or more) combinations. Possible targets are illustrated and summarised in Figure 1. In the present review, we focus on recently emerging data on HMA/VEN-based triplets in AML (excluding acute promyelocytic leukemia), targeting FLT3-, IDH1/2-, and menin-inhibition. In the first part, results from concluded and ongoing clinical trials and real-world experiences on the respective triplet regimen will be reported. Data sources include published, full manuscripts from the past two years. Given the ongoing character of some studies, abstracts from the European Hematology Association (EHA) and American Society of Hematology (ASH) annual meetings from the last two years were also selected. Cited studies include trials in the R/R- as well as in the treatment-naïve setting, with some of them paving the way for advanced phase clinical studies by international consortia and collaborative groups. Their references are highlighted in the respective sections and are indicated separately in Table 1, which provides an overview of the completed and ongoing clinical trials in the corresponding setting.

In the second part of the review, we will discuss general aspects of the triplets. Again, focusing on FLT3-, IDH1/2- and menin-inhibition, open questions with regard to toxicity, post-remission therapy and overall treatment duration are discussed in the context of recently available evidence.

## 2. Novel HMA/VEN-Based Combinations

### 2.1. FLT3 Inhibitors (FLT3i) Combined with HMA/VEN

As highlighted from the molecular profile analysis of the respective benefiting subgroups, the need for improvement for HMA/VEN is particularly high in FLT3-mutated AML [9,10]. FLT3i was approved and successfully used in combination with upfront intensive chemotherapy [27], post-transplant maintenance [28,29] and in the relapsed setting [30]. So, why not combine these inhibitors with the HMA/VEN regimen in order to enhance its efficacy?

FLT3 mutations are late-hit mutations and are often sub-clonal; even if the FLT3 mutated clone is eradicated by a potent FLT3i, generally MRD remains frequently positive due to pre-existing primitive mutations, such as, e.g., WT1, DNMT3A, NPM1 or PTPN11 [31]. The FLT3 mutational status may change at progression or relapse. Resistance to FLT3 inhibition is shown to be multimodal; therefore triplet combinations represent an attractive approach to overcome complex resistance mechanisms [32,33]. Following this hypothesis, AZA/VEN was combined with gilteritinib (GILT) in R/R or treatment-naïve FLT3-mutated AML patients, unfit for intensive chemotherapy [11,12]. Gilteritinib, an oral, second-generation FLT3i, is approved for the treatment of R/R FLT3-ITD or tyrosine kinase domain (TKD)-mutated AML. Patients received the AZA/VEN/GILT triplet in a phase 1/2 trial, consisting of standard AZA (75 mg/m^2^ d1–7), VEN 400 mg d1–28 and GILT 80 mg d1–28 in cycle one. Subsequent dosages were adjusted according to a bone marrow analysis performed on day 14. Responses, as summarized in Table 1, were outstanding with CR rates of 96% in front-line patients, of whom 90% were PCR-MRD negative within four cycles, 50% of them even within two cycles [11,12]. At a median follow-up of 19.3 months, the median relapse-free survival (RFS) in the front-line cohort was not reached. The triplet was well tolerated with a 60-day mortality of 0%. The most common grade ≥3 adverse events (AE) were infections (62%) and febrile neutropenia (38%). In cases of CR, during consolidation cycles, the dosage and duration of each drug were further adjusted/decreased. Treatment continued until disease progression. Motivated by these promising results and other reported retrospective analyses from Lithuania [34], an additional dose-finding and expansion study of the triplet HMA/VEN/FLT3i (mainly GILT or quizartinib) was initiated for ND FLT-mutated AML and is still recruiting (NCT05520567). An interim analysis on *n* = 73 FLT3-ITD or -TKD mutated patients showed an impressive CR rate of 82% with an MRD negativity of 81%, translating into a median OS of 28.1 months [35].

In a phase 2 trial, Maiti and colleagues followed the same concept and exchanged AZA with decitabine (DECI) at 20 mg/m^2^ for 10 days during induction and for 5 days during consolidation [14]. They combined DECI/VEN with GILT or sorafenib in ND (≥60 years) or R/R (≥18 years) unfit AML. Interestingly, among 61% of R/R patients with prior exposure to FLT3i, composite CRc (CR, CRp, CRi) marked 62%, with a negative MRD in all responders. A total of 29% had even prior allogeneic hematopoietic stem cell transplantation (HSCT). When considering only the responding subgroup, MRD negativity by PCR/NGS marked up to 91% in the front-line group and 100% in the R/R group, highlighting the eradicating potential of the DECI/VEN/FLT3i triplet, including in patients with prior FLT3i exposure as well as prior transplant. At 18 months, the progression-free survival in ND and R/R AML was 59% and 58%, respectively [14]. A total of 30% patients were able to reach consolidating allogeneic transplantation (Table 1).

These deep responses were reproduced by the DECI/VEN/quizartinib triplet in a phase 1/2 trial in ND and R/R AML patients [15]. Following this triplet, durable remissions allowed referral to allogeneic HSCT in at least one third of patients, all initially ineligible for intensive chemotherapy (Table 1). These results emphasise the importance of re-evaluating a patient’s fitness throughout the course of treatment. After obtaining deep CR’s by these lower-intensity triplets, patients initially deemed unfit may qualify for more intensive therapies, such as successive allogeneic HSCT consolidation [36,37]. In order to evaluate these preliminary results, a large multi-center phase 1/2 study with AZA/VEN/GILT on ND FLT3 mutated patients is currently recruiting (VICEROY-trial, NCT05520567) [13]. In case of confirmation, these triplet combinations may challenge the current standard for (elderly) FLT3-mutated patients evaluated at diagnosis as ineligible for intensive chemotherapy.

A further development of the DECI/VEN/FLT3i approach is DECI-cedazuridine (ASTX727)/VEN/GILT. The triplet was initially given to FLT3-mutated ND and R/R AML as well as intermediate-2/high-risk myelodysplastic syndrome (MDS) patients [16]. Bataller and colleagues report in a preliminary interim analysis a CRc rate of 83% in the front-line cohort and 44% in the R/R cohort. Of note, 79% and 42% of the cohort had received prior VEN and FLT3i, respectively. A total of 29% were able to proceed to allogeneic HSCT (Table 1). The study is still recruiting and represents a completely novel oral triplet enabling outpatient management. Currently, the European Medicines Agency (EMA) has approved ASTX727 solely for monotherapy in ND AML ineligible for intensive chemotherapy.

Meanwhile, novel FLT3i are arising. Tuspetinib (TUS, HM43239), a multi-kinase inhibitor, targets not only wt and mutant FLT3, but also key pro-survival kinases including SYK, KIT, JAK, RSK2, and TAK1–TAB1, indirectly suppressing MCL1 expression [38]. Pre-clinical data led to the investigation of the TUS/VEN doublet in the global phase 1/2 TUSCANY-trial in (*n* = 79) R/R AML (NCT03850574) [39]. Interestingly, 74% of patients were pre-exposed to VEN, 27.3% to another FLT3i and 26% had previously undergone allogeneic HSCT. Objective responses were observed in the 80 mg TUS arm with a CRc rate of 18.5%, including 30.8% of patients with prior FLT3i exposure. Of note, prior treatment with VEN did not impact their response to the doublet (18.8% and 17.6% in prior VEN-treated and VEN-naïve groups, respectively).

The convincing tolerability of the TUS/VEN doublet encouraged the use of an AZA/VEN/TUS triplet in ND AML patients ineligible for intensive chemotherapy within the same trial (TUSCANY, NCT03850574) [17] requiring similar eligibility criteria as the VIALE-A trial. Early data on 10 patients reported MRD-negative responses within cycle 1, including cases with RAS and TP53 mutations [17,18]. The triplet was generally well tolerated with no significant adverse effects and seemed more active in VEN-naïve patients.

All aforementioned FLT3i were discussed in the context of FLT3-mutated AML, although their influence on FLT3-wt AML is currently under investigation in the randomized, double-blind, placebo-controlled phase 3-QuANTUM-wild-trial following the hypothesis that even FLT3-negative AML may profit from FLT3 inhibition [40,41]. Quizartinib in combination with chemotherapy and as single-agent maintenance is currently tested within 3-QuANTUM-wild on treatment-naïve, FLT3-ITD negative AML.

### 2.2. IDH1/2 Inhibitors Combined with HMA/VEN

Mutations in the isocitrate dehydrogenase (IDH) genes (IDH1 and IDH2) are detected in up to 6–10% of AML patients and play an important role in cell differentiation [42,43]. The 2022 ELN recommendations initially included two oral IDH inhibitors (IDH1/2i), ivosidenib (IVO) and enasidenib (ENA), as monotherapy for salvage treatment in patients ineligible for intensive therapy [44]. A recent long-term follow-up of the AGILE trial highlights a median OS of 29.3 months after AZA/IVO in ND IDH1-mutated patients [42,45]. Of note, the median time to CR achievement of the AZA/IVO doublet was 4.3 months, compared to 1.1 months of the historical AZA/VEN doublet [46]. The longer time to best response is mainly due to its mechanism; IDHi works through the induction of differentiation and not via apoptosis or direct toxicity. Consequently, they risk the on-target adverse effect of a differentiation syndrome, possibly emerging in up to 20% of patients. Their very low myelosuppressive effects make them attractive candidates for combinations in HMA/VEN-based triplets.

An ongoing phase 1b/2 study is investigating AZA/VEN/IVO in R/R and ND IDH1-mutated myeloid malignancies (Table 1) [19,20,21]. Dose level 3 prescribed 400 mg VEN d1–14, 7 days standard AZA and 500 mg IVO p.o. continuously. Doing so, dose-limiting toxicities were G3 tumor lysis syndrome and G3 QT-prolongation, both of which were easily treatable without the necessity of discontinuation. MRD negativity with the triplet was significantly higher than with IVO/VEN, 60% vs. 50%, respectively, underlining the higher potential of the triplet for MRD clearance. In the same study, exploratory analyses on single-cell DNA and protein, as well as time-of-flight mass-cytometry, were performed. No IDH isoform switching nor second-site IDH1 mutations were observed in patients who received the triplet, indicating its potential to overcome resistance pathways, when compared to single-agent IVO [19]. The OS of the entire cohort at a 3-year follow-up was 70.5%, while the event-free survival after 3 years was 50.4 months. However, even if the toxicity of the triple combination was acceptable and the response excellent, the study group was relatively heterogeneous, including both R/R and ND IDH1-mutated AML patients [19,20]. Based on these positive results, a multi-centre, placebo-controlled, phase 3 EVOLVE-1-trial (AMLSG/HOVON) has recently been initiated and will investigate the power of the HMA/VEN-based triplet in ND IDH1-mutated AML patients ineligible for intensive chemotherapy (NCT07075016) [21].

Olutasidenib (FT-2102, OLU), a novel, orally administered IDHi, is reported to exert a specific and particularly tight binding to IDH1 [47,48]. It binds to IDH1 even in the presence of co-mutations that confer resistance to other inhibitors. In the registrational phase 2 trial (NCT02719574) [49], it has shown efficacy in monotherapy (150 mg BID) as well as in combination with AZA [50]. Reported data indicate high response rates and durable remissions with a tolerable side effect profile in R/R IDH1-mutated AML, even in the VEN or IDHi pre-treated patients. In fact, recent U.S. Food and Drug Administration (FDA) approval for R/R IDH1-mutated AML was given, while EMA approval is expected [51]. Moving to the front-line setting, the AZA/VEN/OLU triplet has become the subject of a current single-arm phase 2 trial (NCT06782542).

The IDH2i ENA was combined with AZA and evaluated in an open-label, multi-centre, phase 1b-2 trial (NCT02677922, closed 24/10/24) with AZA alone in ND IDH2-mutated AML. ENA was applied at 100 mg/day or 200 mg/day continuously, in 28-day cycles, combined with standard AZA. The overall response rate (ORR) with the doublet combination was significantly higher than with AZA monotherapy, of 74% to 36%, respectively, *p* = 0.0003. Reported serious treatment-related AEs were comparable in both groups, mainly febrile neutropenia (13% and 16%) and the differentiation syndrome appearing in 10% of AZA/ENA-receiving patients [52]. However, once approved in August 2017 by the U.S. Food and Drug Administration (FDA), the manufacturer’s safety report, for the period of 1 May to 31 July of 2018, reported five cases of deaths associated with a differentiation syndrome in patients who received ENA. Therefore, the FDA currently warns patients and physicians of the need for early recognition and management of differentiation syndrome during AML treatment with IDHi, both ENA and IVO [53]. ENA was never approved by the European Medicines Agency (EMA) and was designated an orphan drug status. Its approval application was retracted (in 2019), underlining that ENA’s toxicity profile requires further clinical investigations with regard to its safety in clinical use.

### 2.3. Menin Inhibitors Combined with HMA/VEN

A lysine methyltransferase 2A (KMT2A) rearrangement is present in around 10% of AML. Menin plays a central role in binding KMT2A proteins and gene promoters [54,55]. Even in NPM1-mutated AML, menin mediates neoplastic transcription [56]. The synergistic apoptotic activity of BCL-2 and menin inhibitors in AML cells has been demonstrated. Firstly, the multi-centric, open-label, phase 1/2 AUGMENT-101-trial investigated revumenib (SNDX-5613, REV) in R/R AML. In a heavily pre-treated population, REV monotherapy showed an ORR of 63.2%, with 22.8% displaying CR, of whom 68.2% were MRD negative (NCT04065399) [57]. AUGMENT-101 is still recruiting and led to the recent approval of REV monotherapy in R/R KMT2A rearranged AML by the FDA. Given its proven efficacy in monotherapy, the following step was to enhance its efficacy by combining REV with the HMA/VEN combination, which was addressed by a completely oral ASTX727/VEN/REV triplet, tested in an initial phase 1/2 SAVE-trial in heavily pre-treated, R/R KMT2A rearranged AML [22,23,58]. This completely oral triplet induced a 58% CR rate, of whom 93% were MRD negative, including patients with prior VEN, prior HMA and even prior HSCT. Of note, the protocol included that patients proceeding to allogeneic HSCT (46%) may partially receive post-transplant REV maintenance for 12 months (Table 1). Based on these promising results, a front-line cohort is now enrolling patients. Despite the small size of the presented cohort, efficacy has been supported recently by Zeidner et al. [24]. They treated ND NPM1+/KMT2A+ patients with the AZA/VEN/REV triplet within a phase 1b Beat AML Master trial (Beat AML, NCT03013998) [24,59]. Two different dose levels were tested in intensive chemotherapy-ineligible elderly patients. Including all patients, CRc rates marked 81%, with all evaluated patients being MRD negative using flow cytometry. Of note, responses were fast with the median time to first response at 28 days; 84% achieved a response within the first cycle. Moreover, a total of 23% of patients were able to proceed to allogeneic HSCT. The study is still ongoing (Table 1). Based on these results, the multi-center, phase 3 randomized EVOLVE-2 (Ho177/AMLSG35-24/UK NCRI, NCT06652438) study is currently launching, assessing AZA/VEN ± REV in adult patients with ND NPM1 or KMT2A rearranged AML ineligible for intensive chemotherapy (Table 1).

A second-generation menin inhibitor, Bleximenib (JNJ-75276617, BLEX), was combined as AZA/VEN/BLEX in R/R and ND AML harbouring KMT2A or NPM1 mutations [25]. A recently presented interim analysis on a total of 120 patients showed high efficacy in R/R as well as in ND patients (Table 1). In treatment-naïve patients, the ORR rate was 92%, with a CRc rate of 85%, without observed differences in response rates between KMT2A-rearranged and NPM1-mutated patients. Most of the commonly reported ≥G3 treatment-emergent adverse events of this triplet were thrombocytopenia (53%), anemia (48%), and neutropenia (46%) with no significant QT-prolongation. A BLEX 100 mg BID dose in the triplet resulted in optimal pharmacodynamic effects [25]. The study is still recruiting. Of note, both menin inhibitors are administered orally. Based on these deep responses with an apparently safe toxicity profile, the HMA/VEN/BLEX triplet will be further investigated within the cAMeLot-2 trial [26], targeting KMT2A-rearranged and NPM1-mutated ND AML patients. Of note, third-generation menin inhibitors such as Ziftomenib, Enzomenib, and Emilumenib are currently under investigation.

### 2.4. Other Novel Drugs Combined with HMA/VEN

The current therapeutic landscape for AML, in both R/R and ND settings, is rapidly evolving. Other possible targets, apart from FLT3, IDH and menin, such as TP53, MCL-1, CD123 and even checkpoint inhibitors, are under investigation in association with HMA, with or without VEN [60,61,62,63,64,65,66,67,68,69,70]. Data on these novel agents are very preliminary and need deeper insights; thus, they will not be addressed in detail. However, some of them are indicated in Table S1 (Supplementary Materials) [60,61,62,63,64,65,66,67,68,69,70].

## 3. Open Questions and Challenges

These findings support the hypothesis that a targeted, multidrug approach may overcome polyclonal disease and therefore obtain deeper responses. The choice of the third agent added to the HMA/VEN backbone is driven by the presence of specific target mutations, including FLT3, IDH, NPM1, and KMT2A. Patients’ cytogenetics and co-mutational status additionally impact treatment response and patient survival [71]. Ongoing phase 3 trials will contribute to a deeper understanding of these effective triplets. However, there are still unmet needs for these triplets, particularly with respect to toxicity management, overall treatment duration, and optimal therapeutic sequencing.

### 3.1. Management of Toxicity

Overlapping myelosuppression of HMA/VEN combinations requires close attention to dosage adjustments and toxicities [72]. A significant limiting factor lies in the length of VEN administration. Multiple studies propose shorter VEN durations for 21, 14 or even only 7 days within the HMA/VEN doublet. DiNardo and colleagues decreased VEN to 21 days in a single-centre phase 2 trial and reported infectious complications only in half of patients, without detracting response rates (84% CR/CRi with 67% MRD negativity) across all ELN-risk groups [73]. Similar results were confirmed by an Indian report on 24 patients treated with a 21-day regimen. In a retrospective study on ND of mainly (66.6%) intermediate/high risk patients, the 60-day mortality of the Indian cohort was 8%, with a CR/CRi rate of 58.3% [74]. In a current Japanese cohort of ND elderly AML patients, Aiba and colleagues indicate that even a 14-day administration of VEN is associated with comparable efficacy and a better safety profile [75]. After decreasing VEN duration from 28 to 21 to 14 days without efficacy reduction, a French group went further and performed a retrospective, multi-centre study with only 7 days of VEN associated with 7 days of AZA in ND, mostly (69.5%) adverse risk AML [76]. Of note, the median time to achieve best response in the cohort was two cycles, similar to the Indian [74] and the DiNardo trials [73]. The median number of cycles to first response was 1 in both groups, while the CR rate was 72% in both groups. Early mortality at 8-weeks was significantly lower with the 7-day VEN doublet compared to the standard dose, 6% vs. 16%, respectively, *p* = 0.03 [76]. Of note, 61% required further dose reduction with the 7 + 7 schedule due to cytopenia. The authors acknowledge the limitations of their retrospective comparison and state that a shortened course of VEN used for 7 days every 28 days resulted in similar response rates and survival when compared to standard VEN exposure.

However, given positive results on different VEN durations with the doublet schedule, extensive evidence originates from a recent, larger, Polish, multi-centric, retrospective analysis of 254 AML patients treated with AZA/VEN. After confirming the VIALE-A results in terms of CRc and OS improvement, Borkun et al. confronted patients with different VEN durations of 14, 21 and 28 days. Patients with 21 days (*n* = 69) or 28 days (*n* = 153) of VEN had a significantly better outcome compared to those who received VEN only for 14 days (*n* = 42), in terms of CRc (69% and 70% vs. 42%, *p* = 0.007) and OS (21 vs. 16 vs. 4.6 months, *p* = 0.005), respectively [77]. Confirmatory data were shown by Ginosyan et al., treating *n* = 85 patients with HMA/VEN in the front-line setting [78]. They compared different VEN durations of 14, 21, and 28 days. The 21-day cohort had the lowest refractory disease rate (14 days: 27.3% vs. 21 days: 14.0% vs. 28 days: 38.7%, *p* = 0.045) and the shortest median time to MRD negativity (14 days: 1.1 months vs. 21 days: 0.8 months vs. 28 days: 1.8 months, *p* = 0.029). Further, compared to 14 days, 21 days of VEN was associated with significantly higher OS of 17.6 vs. 6.1 months (HR 3.08, 95% CI 1.10–8.59, *p* = 0.032) and lower cumulative incidence of relapse (CIR, HR 6.56, 95% CI 2.37–18.2, *p* < 0.001). The 28-day group was comparable with the 21-day group in terms of OS, 15.7 vs. 17.6 months, respectively (HR 1.31, *p* = 0.41), and CIR. Hence, both Bolkun and Ginosyan et al. report inferior outcomes with the shorter 14-day VEN administration [77,78].

These significant results may allow us to reduce doses of the HMA/VEN doublet in case of toxicities. However, the need for dose adjustment investigations becomes more evident with the triplet combinations. Possible options in this context represent: (i) reduction of the VEN dosage as demonstrated with the doublet regimen, (ii) use of granulocyte colony-stimulating factor (G-CSF) support, or (iii) delay of the subsequent cycle based on an early bone marrow blast count, as well as (iv) reduction of the dosage of the other triplet components. The latter approach was applied by Short and colleagues with the HMA/VEN/GILT triplet [11]. In cycle one, they used 7 days of AZA together with 28 days of VEN and 28 days of GILT. However, if the bone marrow on day 14 resulted in <5% blasts or aplastic, then GILT and VEN were stopped, and from cycle 2 on, patients received AZA 75 mg/m^2^ for 5 days, VEN 400 mg for 7 days, and GILT 80 mg for 28 days [11]. In fact, toxicity management of HMA/VEN-based combinations is not standardized yet. In order to evaluate if cytopenia is correlated to toxicity or disease persistence, current NCCN guidelines recommend an early bone marrow aspirate on day 21–28 of the first cycle (NCCN CP Guidelines in Oncology, AML 60–61). In case of CRi, the following cycle may be delayed for 1–2 weeks, G-CSF support can be used, and dose reductions of mostly VEN are discussed [79]. On the contrary, if the bone marrow shows disease persistence, then treatment is recommended to continue without dose reductions or delay. In the VIALE-A trial median time to response was short (1 month). Hence, early response evaluation and dose adjustments based on an early bone marrow analysis seem to represent disease activity accurately.

### 3.2. Post-Remission Treatment

Another aspect of toxicity relies on the overall treatment duration once CR has been achieved. Despite AZA/VEN-based regimens representing continuous treatments, initial data about discontinuation of the doublet are emerging. Chua and colleagues presented an Australian retrospective analysis of 29 ND AML patients in remission for a minimum of 12 months on treatment [80]. A total of 55% continued therapy until disease progression, while 45% electively stopped (STOP). With a more than 5-year follow-up, they reported a median treatment-free remission time of 45.8 months among the STOP cohort. More than 50% of patients sustained remission. Of note, the risk of relapse, the duration of RFS, and the OS were similar between the two cohorts. Confirmatory data were presented from the French Innovative Leukemia Organization (FILO) Centers and the Moffit Cancer Center (US) [81]. In a total of 62 ND patients, 34 continued AZA monotherapy, while 28 patients discontinued due to prolonged cytopenia, non-hematological toxicities, patients’ choice or unknown reasons. Patients who stopped treatment displayed similar outcomes compared to those continuing. Interestingly, compared to the Australian study [80], in the French study [81], the prerequisite of being in remission for at least a year was not even required. Nevertheless, data indicate that not only a discontinuation of the AZA/VEN doublet may be feasible, but in case of relapse after discontinuation, a re-challenge with AZA/VEN may be seriously considered. In this context, only IDH mutations and an R/R status are statistically significant for OS in multivariate analysis, not the discontinuation itself [82].

With respect to some triplet regimens, a significant proportion of patients also did not continue. After two of the FLT3i-containing [14,16] and two of the menin-inhibitor-containing [22,24,59] triplets, initially intensive therapy-ineligible patients were able to proceed successfully to allogeneic HSCT. This aspect does underline the less-intensive character of the regimen and supports the stability of their—in part MRD-negative—responses. After achieving CR with the triplets, eligibility for intensive treatment may improve. However, the question arises: is the outcome with allogeneic HSCT after these HMA/VEN-based regimens comparable with that of allogeneic HSCT following intensive chemotherapy? Senepati and colleagues compared and responded that treatment with an HMA/VEN-based, less intensive regimen (*n* = 35) was associated with favourable outcomes in older patients who underwent subsequent allogeneic HSCT [83]. The OS was comparable to that with intensive chemotherapy (*n* = 42), followed by allogeneic HSCT, even with a CIR rate significantly lower. Interestingly, their regimen consisted of a quadruplet, administered by an alternating approach: two cycles of HMA/VEN alternated with two cycles of cladribine and cytarabine. This schedule represents an additional way of drug composition: components were not administered simultaneously, but in an alternating manner. A total of 93% of patients responded to this HMA/VEN-based alternating quadruplet and 84% were negative for measurable MDR [84]. The multi-centric study is still ongoing and currently recruiting (NCT03586609).

### 3.3. Limitations of the HMA/VEN-Based Triplet Studies

Most of the triplet trials are performed on small patient subsets due to the rarity of the investigated genomic aberration and the required expertise only being available in a limited number of centres. Given the pivotal character of most reported triplet trials, treatment-naïve and R/R AML patients were included, leading to heterogeneous patient cohorts. With regard to the R/R patients, the aspect of VEN, HMA or, e.g., FLT3i pre-treatment represents an important factor. The paradigm of avoiding an agent in subsequent treatment lines seems to be challenged by the triplet concept. Different class inhibitors are available and seem to induce significant responses, even in heavily pretreated patients. Patients previously exposed to VEN and the target inhibitor are reported to show responses [14,16,39]; whether these responses are based on the triplet’s capacity of overcoming treatment resistance is not clear, although it is hypothesized [32,33]. Treatment sequencing is arbitrarily approached in the reported trials. However, a significant portion of patients consolidated their response to the HMA/VEN-based triplet with allogeneic HSCT [14,16,19,58], with some of them even consequently maintaining the mutation targeting agent [58]. Neither the impact on graft versus leukemia, graft versus host disease, nor the impact on toxicity and final OS is currently specified in the cited works. Of note, the majority of the general toxicity issues, e.g., in the case of VEN duration, are based on the HMA/VEN doublet experience. However, most of the cited trials are very recent and ongoing; their broader toxicity data, as well as longer follow-ups, may lead to developments of the correct dosage of the respective components within the respective HMA/VEN-based triplet.

## 4. Conclusions

The very recent implementation of the HMA/VEN combination regimen into routine clinical practice promises to change completely our historical treatment approach of AML patients ineligible for intensive chemotherapy. Outstanding results have led to new ELN 2024 recommendations, based on genetic risk classification [85]. Currently arising, respective triplet combinations are tailored to the patients’ mutations and show higher response rates than the HMA/VEN doublet alone. Their significant efficacy is not only documented in (heavily) pre-treated but also in ND AML, mimicking the results of standard intensive chemotherapy. Indeed, currently ongoing clinical trials aim to compare the triplets with standard intensive chemotherapy in ND, young patients. Further, fully oral triplet regimens based on the oral DECI formulation with VEN and an FLT3, IDH or menin inhibitor are currently under investigation. Deep MRD responses allow to improve transplant eligibility in a subset of patients. Nevertheless, there are many unmet challenges, such as management of toxicity with the respective triplet, timing of response assessment including MRD monitoring, co-mutational disease status, treatment resistance and duration, just to name a few.

To address these gaps, consortia and networks, such as the EVOLVE Steering Group (AMLSG, HOVON and UK AML Clinical Trial Group) and the Key EVOLVE Collaborative Groups, are arising. The initiation of advanced phase, multicentric, clinical trials by these societies (Table 1) will validate the efficacy of HMA/VEN-based triplets in different hematological centres. In case of positive results, they may change our historical approach, not only in R/R, but also in ND, elderly and young AML. By doing so, recent guidelines may potentially necessitate significant updates.

## Figures and Tables

**Table 1:** HMA/VEN-based triplets in newly diagnosed and relapsed/refractory AML: Data are indicated as *n* (%) or median. CR: complete remission; CRi: complete remission with incomplete recovery; MRD^neg^: minimal residual disease negativity, * indicates MRD negativity by PCR/NGS; OS: overall survival; mo: months; AZA: azacytidine; VEN: venetoclax; 1.line: first line; R/R: relapsed/refractory; NR: not reached; DECI: decitabine; GILT: gilteritinib; SORAF: sorafenib; QUIZ: quizartinib; TUS: tuspetinib; IVO: ivosidenib; OLU: olutasidenib; REV: revumenib; BLEX: bleximenib; allo HSCT: consolidation with allogenic hematopoetic stem cell transplantation after experimental treatment.

TARGET	TRIPLET	TRIAL	PHASE	PATIENTS (*n*=)	CR/CRi (%)	MRD^neg^ (%)	MEDIAN OS (mo)	ALLO HSCT (%)	INITIATED in 1.line (Phase)
FLT3	AZA/VEN/GILT	NCT04140487 Recruiting [11,12]	1/2	1.line 30 R/R 22	96 27	90 * 43 *	29.7 5.8	47 23	NCT05520567 “VICEROY” (1/2) [13]
	DECI/VEN/ SORAF or GILT	NCT03404193 [14]	2	1.line 12 R/R 13	92 62	91 * 100 *	NR 6.8	30 38	
	DECI/VEN/QUIZ	NCT03661307 Recruiting [15]	1/2	1.line 19 R/R 47	95 60	82 * 29 *	NR 6.3	26 34	
	Cedazuridine/ VEN/GILT	NCT05010122 Recruiting [16]	1/2	1.line 7 R/R 7	83 44		NR 6.8	29	
	AZA/VEN/TUS	NCT03850574 Recruiting [17,18]	1/2	1.line 10	90	56			NCT03850574 “TUSCANY” (1/2) [17]
IDH1	AZA/VEN/IVO	NCT03471260 Recruiting [19,20]	1b/2	1.line 14 R/R 8	93 63	60 * 50 *	NR 9		NCT07075016 “EVOLVE-1” (3) [21]
	AZA/VEN/OLU	NCT06782542 Recruiting	2	1.line					
MENIN	Cedazuridine/ VEN/REV	NCT05360160 Recruiting [22]	1/2	R/R 26	58	93	NR	46	NCT05360160 SAVE (1/2) [23]
	AZA/VEN/REV	NCT03013998 Recruiting [24]	1b	1.line 43	81	100	15.5	23	NCT06652438 “EVOLVE-2” (3)
	AZA/VEN/BLEX	NCT05453903 Recruiting [25]	1b	1.line 34 R/R 86	85 54				NCT06852222 “cAMeLot-2” (3) [26]

## Data Availability

Given the nature of a review, all cited data are publicly available.

## Supplementary Materials

The following supporting information can be downloaded at: https://www.mdpi.com/article/10.3390/cancers18040615/s1, Table S1: HMA/VEN based regimens in newly diagnosed AML except FLT3-, IDH and menin inhibitors.
