# Supplementary material for: Hypomethylating Agents and Venetoclax Based Triplets Targeting FLT3, IDH and KMT2A in Acute Myeloid Leukemia: Current Studies and Challenges of a Tailored Approach
Source: Cancers (Basel). 2026 Feb 13;18(4):615. doi: 10.3390/cancers18040615 (PMC12939284; doi:10.3390/cancers18040615)
Supplement: Supplementary file 1 [file cancers-18-00615-s001.zip › cancers-4104183-supplementary.pdf]

TARGET	ASSOCIATION	TRIAL PHASE	PATIENTS (n=)	CR/CRi (%)	MEDIAN OS (mo)	REFERENCE
**CD47/TP53**	AZA/VEN/MAGRO	1b/2	17	12	3.1	NCT04435691[71]
	AZA/VEN/MAGRO	3	189	39.7	11.7	NCT05079230[72]
**TP53**	AZA/EPRE	1b/2	11	36	10.8	NCT03072043[73]
	AZA/VEN/EPRE	1	49	38	n.a.	NCT04214860[74]
**CD123**	AZA/TAG ± VEN	1b	34	89	n.a.	NCT03113643[75]
	AZA/TAG ± VEN	1b	26	39/19	14	NCT03113643[76]
**NEDD8-NAE**	AZA/VEN/PEVO	1/2	40	50/13	8.1	NCT03862157[77]
**Hedgehog**	AZA/GLAS	3	322	19.6	10.3	NCT03416179[78]
	LDAC/GLAS	2	132	18.4	8.3	NCT01546038[79]
**MCL-1**	AZA/VEN/HOMO	2	96	70.8	22.1	NCT04424147[80]
**PD-1/PD-L1**	AZA/NIVO	2	70	22	-	NCT02397720[81]

Supplementary Table S1. HMA/VEN based regimens in newly diagnosed AML except FLT3-, IDH and menin inhibitors. Data are indicated as n (%) or median. CR: complete remission; CRi: complete remission with incomplete recovery, OS: overall survival; AZA: azacytidine; VEN: venetoclax, DECI: decitabine; MAGRO: magrolimab; n.a.: not available; EPRE: Eprenetapopt; TAG: tagraxofusp; LDAC: low dose cytarabine; PEVO: pevonedistat; GLAS: glasdegib; HOMO: homoharringtonine; NIVO: nivolumab; LDCA: low-dose cytosine arabinoside
